# Assessing factor structure and reliability of the career adaptability scale in students with special educational needs

**DOI:** 10.3389/fpsyg.2023.1030218

**Published:** 2023-02-15

**Authors:** Lan Yang, Kuen Fung Sin, Mark L. Savickas

**Affiliations:** ^1^Department of Curriculum and Instruction, The Education University of Hong Kong, Tai Po, Hong Kong SAR, China; ^2^Analytics\Assessment Research Centre (ARC), The Education University of Hong Kong, Tai Po, Hong Kong SAR, China; ^3^Centre for Special Educational Needs and Inclusive Education (CSENIE), The Education University of Hong Kong, Tai Po, Hong Kong SAR, China; ^4^Department of Special Education and Career Counselling, The Education University of Hong Kong, Tai Po, Hong Kong SAR, China; ^5^Department of Family and Community Medicine, Northeast Ohio Medical University, Rootstown, OH, United States

**Keywords:** career adaptability, career development, CAAS-SF, SEN students, career guidance, life planning, Chinese students, inclusive education

## Abstract

Despite the importance of career guidance and life planning education in helping students’ career development, considerably limited research has been done to provide a good educational assessment to identify SEN students’ strengths and weaknesses of career adaptability. This study aimed to assess the factor structure of the career adaptability scale in mainstream secondary students with special educational needs. The results support adequate reliabilities of the total scale and subscales of the CAAS-SF among over 200 SEN students. The results also support the four-factor structure of the career adaptability construct in assessing career concern, control, curiosity and confidence. We also found its measurement invariance across gender at the scalar invariance level. The positive and significant correlation patterns between boys’ and girls’ career adaptability and its sub-dimensions with self-esteem are similar. Overall, this study support that the CAAS-SF is a good measure with adequate psychometric properties for assessing and developing practical career guidance and life planning activities and programs for SEN students to support their career development needs.

## Introduction

Career adaptability is essential in personal career preparation and development (Savickas, 2012). It refers to individuals’ capabilities to prepare for predictable tasks involved in job roles and to adjust accordingly to unpredictable factors arising from changes in working conditions (Savickas, 1997, 2012). According to career development theory (Super and Knasel, 1981), career adaptability is integral to career development. However, it is different from other career-related factors, such as career decision-making self-efficacy and career choice commitment (Duffy and Blustein, 2005), career outcome expectations (Kenny and Bledsoe, 2005), and career exploration (Zikic and Klehe, 2006). Savickas (1997) recommends that self-development and career adaptability include several aspects: exploring available opportunities, planning for the future, making appropriate choices, and managing all relevant personal and environmental factors to achieve one’s career-building and life-planning goals ultimately. Since the 1990s (Savickas, 1997), the notion of career adaptability has drawn increasing attention in examining individuals’ career development. Career adaptability has gradually developed into a multidimensional psychosocial resource that can help individuals manage career-related tasks and career transitions during the work period (Savickas, 2002, 2005, 2012). According to Savickas's (2002) career construction theory, as a psychosocial construct, career adaptability has four dimensions: concern, control, curiosity, and confidence. Concern refers to an individual’s attention and preparation for the future; Control refers to an individual’s responsibility for the future; Curiosity refers to an individual’s exploration of multiple career options for their future and the possibilities that affect their career choices; Confidence refers to an individual’s self-perception of their ability to achieve their career goals (Savickas and Porfeli, 2012). These dimensions work jointly to form “an individual’s resources for coping with current and anticipated tasks, transitions, traumas in their occupational roles” (Savickas and Porfeli, 2012, p. 662). Researchers also found career adaptability is significantly associated with adapting responses (e.g., career exploration, career decision-making self-efficacy) and adaptation results [e.g., employability, career identity, career/school satisfaction; see details in the meta-analysis conducted by Rudolph et al., 2017a based on over 90 studies].

However, up to the present, research on career adaptability among students with special educational needs appears scarce. With 109 senior secondary school SEN students and a matching sample of typically-developed students, Yang et al. (2020) examined the measurement invariance of the Career Development Self-Efficacy Inventory (CD-SEI; Yuen et al., 2005) in SEN students to assess five dimensions of CD-SEI: training selection, career goal setting, career planning, job-hunting preparation, job hunting. The results supported the metric invariance of CD-SEI between SEN and typically-developing students, suggesting this scale measures the invariant factor structure between the two groups of students. Although the CD-SEI tested appropriately the five dimensions, career adaptability was not included. The below sections elaborate on our comprehensive literature review on career adaptability to highlight a compelling need to prepare a suitable measure of career adaptability for SEN students.

## Literature review

### Career construction theory: A snapshot

Career construction theory, according to (Savickas, 2013), posits that individuals have different roles across lifetime. They may initially form a social role composed through their actions in their families, extend this role to schools and communities, and continuously expand and adapt this role to their occupations to form narrative stories of career identity and adaptability. Precisely, Savickas (2013) commented career construction theory “attends to an individual’s behaviors as an actor, strivings as an agent, and explanations as an author” (p. 147). The role of being an actor has its objective perspective of displaying individuals’ stories ranging from their schooling to retirement. The role of being an agent focuses on individuals’ strivings and adaptations pursued in various occupations. The role of being the author has its projective feature of examining individuals’ reflections on their work experiences to generate meaningful career stories (also known as the autobiographical reflexivity of individuals’ own life stories). From a developmental perspective, career construction theory describes the three interrelated and progressive roles (actor, agent, author) individuals perform across lifetime. In the process, Savickas introduced adaptability and identity as two competencies individuals need to equip themselves for career construction in the 21st century.

### Career adaptability

Based on career construction theory, researchers (Savickas, 2013; Fiori et al., 2015) suggested that individuals with high career adaptability are more likely to have smooth career transitions and establish appropriate associations between themselves and their careers. With adolescents as samples, researchers found that those with higher career adaptability are more likely to succeed in post-school transitions (Germeijs and Verschueren, 2007; Skorikov, 2007). Most studies have shown that career adaptability is positively correlated with an individual’s life, such as positive attitudes toward the future (e.g., Ginevra et al., 2016, 2018) and life satisfaction (Hirschi, 2010). Hirschi (2010) investigated the impact of career adaptability on secondary student development and predictors of life satisfaction among 330 Swiss eighth-grade students. The results showed that increased career adaptability over time significantly positively impacted youth development and life satisfaction. In another longitudinal study, Marcionetti and Rossier (2021) examined the reciprocal relationship between career adaptability and self-esteem among 437 junior secondary students. The results supported the reciprocally mutual reinforcing effects of career adaptability and self-esteem on each other. Marcionetti and Rossier's (2021) study also suggested enhancement interventions on changing adolescents’ self-esteem of being able to cope with different life events would lead to improved career adaptability and vice versa. With a sample of university students, Guan et al.’s (2017) longitudinal study also found the predictive effects of personal traits (e.g., Big-five personality) on career adaptability and the mediating effect of core self-evaluation on the relationship between traits and career adaptability, suggesting positive changes of these predictors would enhance career adaptability.

In recent years, many studies on career adaptability have contributed empirical data to meta-analysis research. For example, some scholars conducted a meta-analysis of 92 independent studies (Rudolph et al., 2017a). The key finding was that career adaptability was positively correlated with career planning, career exploration, and decision-making self-efficacy and significantly impacted subjective well-being. Subjective well-being (e.g., life satisfaction) also positively affected career adaptability. Rudolph et al.’s (2017a) study also presented the effects of career adaptability on a wide range of career-related variables, including career identity, career satisfaction, job satisfaction, school satisfaction, affective organizational commitment, normative organizational commitment, ongoing organizational commitment, employability, job performance, income, engagement, entrepreneurial outcomes, and life satisfaction. In another meta-analysis study by Rudolph et al. (2017b), these researchers analyzed 76 independent studies (total sample of 39,543 people) to examine the four dimensions of career adaptability (concern, control, curiosity, and confidence), as well as the relationships between these four dimensions and other outcome variables such as job performance, job satisfaction. Rudolph et al. (2017b) found that the control dimension of career adaptability was positively associated with job and life satisfaction, while confidence was strongly associated with school satisfaction and employability. Overall, the two meta-analyses collectively contributed to a general picture of research development of career adaptability and its influencing factors. However, these two studies have not paid attention to the group attributes, such as the group of students with disabilities. Johnston's (2018) systematic review screened 116 published studies in more comprehensive sources (book chapters, articles) and research designs (e.g., cross-sectional, longitudinal, intervention, and theoretical) than previous meta-analytic studies. The scholar introduced existing measures for career adaptability and highlighted “future research needs to address these measurement challenges” including further assessment of adaptability resources and responses (Johnston, 2018, p. 19). As for theoretical suggestions, Johnston found the prevalence of career construction theory in supporting research development of the career adaptability (CA) construct (CCT; Savickas, 2013). We have added one paragraph to introduce CCT for *CA.* In these 116 studies, Johnston (2018) found solely one study assessing career adaptability among adult workers with intellectual disabilities Santilli et al. (2014). We will elaborate on Santilli et al.’s (2014) work in the below section. Key findings of these meta-analytic and systematic reviews showed that research on career adaptability among students with special educational needs remains understudied.

### Post-school transition situations and adaptability of students with disabilities

Previous studies have found that unemployment or underemployment often happens to secondary school students with disabilities during the transition from high school to adulthood (Horvath-Rose et al., 2004; Sin and Yang, 2018). Their unique disability experiences may ultimately affect their Career adaptability (Fabian and Liesener, 2005). Therefore, in previous studies, some researchers suggested that more research be done to understand the career adaptability of this group, and pointed out the importance of providing these people with career adaptability skills training, such as training in problem-solving skills, communication skills or time management competence (Lindstrom et al., 2013; Yang et al., 2015). It is worth noting that the research review found that the number of career adaptability surveys for adolescents with disabilities is scarce. Limited research has investigated career thinking and attitudes. For example, Dipeolu et al. (2013) examined the effects of career thoughts and attitudes on the vocational identity of high school students among 119 high school attention-deficit/hyperactivity disorder students from the United States. The results supported career thoughts and attitudes as two valuable predictors. Dipeolu et al. (2013) also suggested that students with ADHD or other types of disabilities experienced more difficulties in career adjustments due to disability-related issues during the career counseling process and called for proper career-related measures to assess and improve the career decision-making process of ADHD students. Another study by Santilli et al. (2014) examined the relationship between career adaptability, hope, and life satisfaction in 120 adult workers with mild intellectual disabilities using a partial mediation model. The results show that career adaptability is directly or indirectly related to individual life satisfaction. A recent study in Hong Kong (Yuen and Chan, 2022) examined the predictive effect of social connectedness on the presence of meaning in life, career adaptability, and self-efficacy of 267 SEN students (the majority of participants were boys from grade 7).

The results showed that meaning in life significantly mediated the relationship between social connectedness and two career outcome variables (adaptability and self-efficacy). Career adaptability was also positively associated with career self-efficacy. However, the research scope of Yuen and Chan's (2022) study was not to validate the career adaptability scale in SEN students. Instead, it was treated as a general factor in their SEM analysis. Given this, detailed psychometric properties of the CAAS-SF among the Grade 7 SEN students remain unknown. Furthermore, the two scholars focused on mainly three major types of SEN: “attention-deficit/hyperactivity disorder (*n* = 114, 33.0%), reading/writing difficulties (*n* = 102, 29.6%), and speech/language difficulties (*n* = 58, 16.8%)” (Yuen and Chan, 2022, p. 8). Given that the types of SEN students integrated into regular classes have reached nine types^1^ in Hong Kong (Education Bureau, 2021), for more implications of validating an instrument, it would be more meaningful for researchers to include more SEN types than only three and more grades than only grade 7 in further research.

The above literature review on career adaptability shows that: (a) the research on groups with special educational needs is exceptionally scarce; (b) the surveys on the career adaptability of middle school students with disabilities are mostly untargeted, only to investigate the variables related to it (e.g., career maturity); (c) most of the studies focus on general populations of students in secondary and higher education to explore their career adaptability; (d) career adaptability have significant impacts on individual career development, life quality, and satisfaction, especially for those with disabilities. However, research on the career adaptability of students with special educational needs integrated into mainstream classrooms remains scarce.

### Measurement of career adaptability

To assess career adaptability, Savickas and Porfeli (2012) developed the Career Adaptability Scale (CAAS, 24 items), which consists of four dimensions: concern, control, curiosity, and confidence. The 24-item version of CAAS has been validated in 13 countries/regions through an international collaborative survey, and the results show that CAAS has high reliability and validity across countries (Savickas and Porfeli, 2012). Moreover, career adaptability also has predictive validity. Research has shown that career adaptability is related to demographic characteristics such as age, gender, education (Hou et al., 2012), and nationality (Dries et al., 2012). Hou et al. (2012) found that male college students are more concerned than female college students of the same age, and the career adaptability of senior college students is lower than that of first-year students. Cross-cultures studies also found that career adaptability and personality traits are associated with participants from Brazil, Belgium, and Switzerland (Rossier et al., 2012; Teixeira et al., 2012; van Vianen et al., 2012). For example, personality traits such as extroversion, conscientiousness, helpfulness, and openness to experience are positively associated with Career adaptability and neuroticism (Teixeira et al., 2012). Career adaptability is also positively correlated with personal interest, well-being, and quality of life but negatively correlated with stress and life barriers (e.g., Porfeli and Savickas, 2012; Soresi et al., 2012). Compared with personality traits, career adaptability is a stronger predictor of individual work engagement, and career adaptability also moderated the impact of personality on work engagement (Rossier et al., 2012). Some interview studies have found that career adaptability interacted with and influenced job transition (e.g., Brown et al., 2012; McMahon et al., 2012). Using grounded theory, McMahon et al.’s (2012) qualitative study found that career adaptability manifested in different life contexts among older women (aged 45 to 65 years). These women differed qualitatively across five dimensions of career adaptability (concern, control, curiosity, confidence, and collaboration).

While the above paragraphs introduced critical findings of studies included in the special issue of the Journal of Vocational Behavior measuring career adaptability across 13 countries/regions, only one study surveyed 296 mainland Chinese college students (Hou et al., 2012). Given the popularity of the CAAS in assessing career adaptability worldwide, researchers also further explored possibilities to shorten it to explore its relationship with more variables to be evaluated by other scales. To facilitate the integration of the CAAS into extensive surveys (Maggiori et al., 2017), developed a short form with 12 items (CAAS-SF) to assess the four dimensions of career adaptability among over 2,000 French and German-speaking participants in Switzerland. Yu et al. (2020) tested the CAAS-SF across three samples of Chinese participants: colleague students, civil servants, and enterprise employees. Reliabilities ranged from 0.77 to 0.88 for the four dimensions and 0.91 to 0.94 for the total scale, showing the approrateiness of this short form as a reliable measure of career adaptability of Chinese participants.

Seeking a general picture of the implementation of CAAS in students with special educational needs, we screened among the 267 journal articles published in SSCI journals documented in the Web of Science (WoS), and 82 were published by Chinese scholars (30%). Among the 82 studies, 24 used students as the main subjects (*N* = 16,987), of which most were students in vocational colleges and universities with only three studies (Yuen and Yau, 2015; Xu et al., 2020; Datu and Buenconsejo, 2021) that aimed at middle school students (*n* = 1,239). Yuen and Yau (2015) examined the relationship between 543 Grade 9 Hong Kong students’ career adaptability, meaning in life, and connectedness to school. The results showed that connectedness to school and meaning in life have different predictive effects on the four dimensions of career adaptability. The predictive relationships also differ in male and female students. Xu et al. (2020) explored the direct and indirect predictive effects of career adaptability on mental health problems of 372 Chinese high school students. The results showed the directly negative predictive effect of career adaptability on mental health problems, suggesting high career adaptability may lead to fewer mental health problems. Resilience was also found as a significant mediator that negatively mediated the relationship between career adaptability and mental health problems. Xu et al. (2020) argued that “resilience may be conceptualized as an adaptive process to cope with career development tasks and changing work and career conditions” (p. 4). However, Xu et al.’s (2020) study did not examine the four dimensions of career adaptability but regarded this variable as a general factor. Datu and Buenconsejo (2021) examined the predictive effects of academic engagement and achievement on the career adaptability of 324 Filipino high school students. They found behavioral engagement appears to be the strongest predictor of the four dimensions of career adaptability compared to emotional engagement, academic achievement (the weakest predictor), and other demographic variables (e.g., age and gender).

As the review results elaborated above show, there is a lack of career adaptability research in SEN students. Sin and Yang's (2018) study would be one of the pioneering studies to explore the post-school transition of SEN students with a wide range of stakeholders in Hong Kong: 73 SEN students, 37 SEN students’ parents, 64 school teachers, and 15 social workers. The two scholars summarized key qualitative findings as follows: “(a) students with SEN have limited choices for further education and post-school employment; (b) parents of students with SEN consistently reflected their high expectation on SEN students’ further education; (c) available career-related guidance and activities have not been tailored to suit SEN students’ diverse needs; (d) home-school partnership in supporting the SEN students’ post-school transition was weak” (Sin and Yang, 2018, p. 191). Despite its contribution, its apparent limitation was the non-involvement of career adaptability measures. In a recent study by Salimi et al. (2022), these scholars examined the relationship between career satisfaction and career adaptation among 319 adult participants with visual impairment. The result showed a significant predictive effect of career adaptability on career satisfaction. However, Salimi et al. (2022) did not examine the factor structure and the four dimensions of career adaptability. In a recent review conducted by Wong (2022), this scholar reviewed 463 published studies on career adaptability in a range of disciplines of psychology and found that “most published studies in Hong Kong on career adaptability did not use data and methodological triangulation research methodologies” (p. 181).

## The present study

Given a lack of studies on measuring career adaptability of disabilities, the key aim of this study was to extend previous empirical work on examining the Career Aapt-Abilities Scale in Chinese students (e.g., Hou et al., 2012; Yuen and Yau, 2015) to students with special educational needs in Hong Kong. In this study, we use ‘special educational needs (SEN)’ to refer to children with learning disabilities instead of using ‘disabilities’ directly to replace SEN. Doing so is consistent with the term used by the Educational Bureau of the Government of Hong Kong SAR (source https://www.edb.gov.hk/en/curriculum-development/curriculum-area/special-educational-needs/index.html) and international use of SEN. The key research objective was to assess the factor structure of the CAAS by comparing its internal construct with one general factor (career adaptability as a whole) and with multidimensional factors with a sample of students with SEN students in Hong Kong mainstream education.

### Participants and procedure

We recruited 204 students with special needs (131 boys and 70 girls, three missing gender information) from 13 mainstream secondary schools. Among them, 40.2% were from Grade 7, 41.2% from Grade 8, and 18.6% from Grade 9. Their mean age was 16.36 (SD = 1.22; ranging from 14 to 21), with 14 (6.9%) missing age information. One hundred six students identified with one type out of the nine types of special educational needs. Specific sample distribution is as follows: Specific Learning Difficulties (*n* = 46), Attention Deficit/Hyperactivity Disorder (*n* = 21), Autism Spectrum Disorder (*n* = 13), Mental illness (*n* = 10), Physical Disability and Visual Impairment (*n* = 5), Hearing Impairment (*n* = 2), Speech and Language Impairment (*n* = 4), Intellectual Disability (*n* = 1). Forty students (19%) have two SEN types, and 31 (15%) have more than two SEN types. Twenty-seven students (14%) did not report their SEN types.

We achieved ethical approval from the Human Research Ethics Committee of the leading author’s university before this survey study. Before completing the survey, we collected consent forms from all participants and their parents (as guardians) in this study. We clearly informed SEN students and their parents about the purpose of this study and their/their children’s freedom to withdraw from filling out the questionnaire without any negative consequences. A research assistant trained for this project and invited local school teachers to assist the research team in distributing hard copies of the questionnaire and collecting data from the targeted schools.

### Measures

The Career Adaptation Scale-Short Version (CAAS-SF, 12 items) was validated by Maggiori et al. (2017) based on Savickas and Porfeli (2012). This study translated the CAAS-SF into Chinese by filling the translation-back translation procedure (Chang et al., 1999; Phongphanngam and Lach, 2019) by three bilingual researchers of this study. The CAAS-SF assessed participants’ strength level of career adaptability which refers to students’ preparation state and adaptability resources needing for current and future-oriented career development tasks. Career adaptability consists of four subscales: Concern (individuals’ plans for their future-oriented career tasks; a sample item is “Preparing for the future.”), Control (individuals’ responsibility taking for personal career development; a sample item is “Making decisions by myself.”), Curiosity (individuals’ envisions for future selves in the workplace environment; a sample item is “Observing different ways of doing things.”) and Confidence (individuals’ perceived self-ability to succeed in careers; a sample item is “Working up to my ability”). Each scale consists of three items. Students rated on a five-point scale ranging from 1 = strongly disagree to 5 = strongly agree. In this study, reliabilities of the four dimensions of CAAS-SF (concern, control, curiosity and confidence) are 0.91, 0.80, 0.84, 0.68 for the total sample of SEN students, 0.94, 0.81, 0.84, 0.72 for boy SEN students and 0.87, 0.77, 0.83, and 0.59 for girl SEN students. Reliabilities of the whole scale of CAAS-SF (12 items) for the total sample, boys and girls, are 0.91, 0.93, and 0.88, respectively.

We used a short self-esteem scale (3 items, Rosenberg et al., 1995) to explore the external validity of the CAAS-SF. Self-esteem refers to one’s global sense of self-worth as a person (Rosenberg, 1979; García et al., 2019). Given that the four dimensions of career adaptability were measured from a perspective of students’ self-perceptions and self-reports, self-esteem as a broad self-perception of one’s self-worth would have positive and significant correlations with concern, control, curiosity, and confidence measured by the CAAS-SF. RSES has been demonstrated to be the most used measure of self-esteem across over 50 nations (e.g., Schmitt and Allik, 2005; Gnambs et al., 2018) and different populations, including students with disabilities (Syropoulou et al., 2021). We used the Chinese version of the Rosenberg Self-Esteem Scale (RSES, Wu et al., 2017) for assessing SEN students’ self-esteem. In addition, the short form of RSES has also been examined and found with good psychometric properties (Monteiro et al., 2022). We used positive-worded items instead of mixing them with negative-worded items based on suggestions in previous research. For example, Lindwall et al. (2012) found “in many cultures the answers to negatively worded items [of RSES] are systematically different from the answers to positively worded items” (p.197). The three self-esteem items are “On the whole, I am satisfied with myself.,” “I feel that I’m a person of worth, at least on an equal plane with others.,” and “I am able to do things as well as most other people.” Consistent with the CAAS-SF, SEN students rated on a five-point rating scale ranging from 1 = Strongly disagree to 5 = Strongly agree. In this study, reliabilities for the total sample, boys and girls with SEN are 0.82, 0.85, and 0.78, respectively.

### Statistical analysis

#### Within network validity

**Figure 1:**
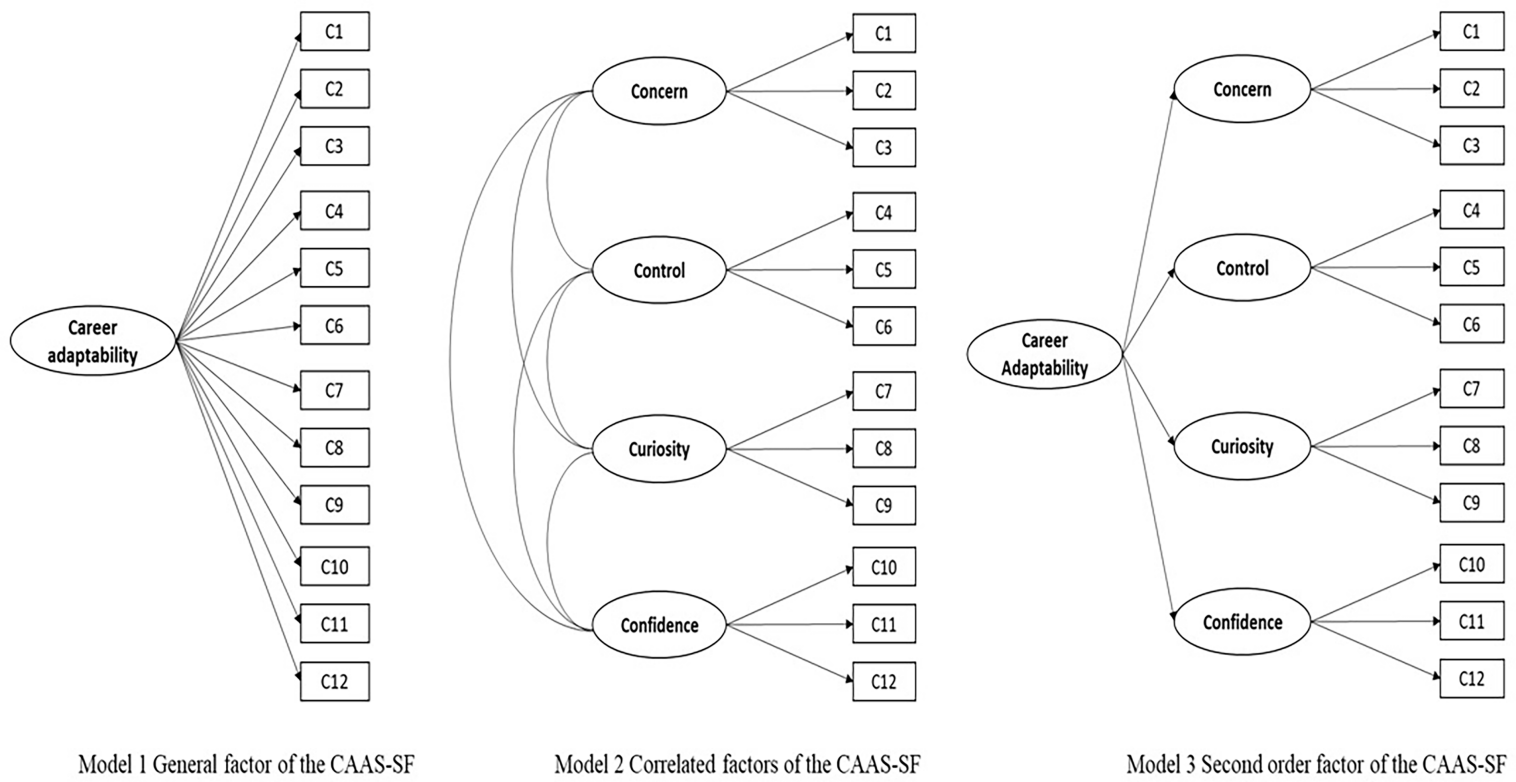
Three alternative models of CAAS.

To examine the within-network validity of the CAAS-SF, we performed a series of confirmatory factor analyses using *Rstudio* and the lavaan package. We tested three alternative models to explore the factor structure of the CAAS-SF with MLR estimator (maximum likelihood estimation with robust standard errors). The three models, as shown in Figure 1 are as follows: a single-factor model with all items loading on one factor, a four correlated factors model, a second-order model with four first-order factors, and 1 s-order factor. Model fit was evaluated using the comparative fit index (CFI), Tucker–Lewis index (TLI), root mean square error of approximation (RMSEA), and standardized root means square residual (SRMR). CFI and TLI values higher than 0.90, RMSEA and SRMR values lower than 0.08 indicated acceptable model fit; CFI and TLI values higher than 0.95, RMSEA and SRMR values lower than 0.06 suggested good fit (Hu and Bentler, 1999).

#### Measurement invariance across gender

Further, measurement invariance across gender was tested by using a hierarchical set of equality constraints over gender groups. Specifically, we tested the configural invariance (freely estimating all parameters), metric measurement invariance (constraining factor loadings), scalar measurement invariance (constraining factor loadings and item intercepts were constrained to be invariant across groups), and residual measurement invariance (constraining factor loadings, item intercepts, and item uniqueness). Invariance was evaluated using the changes in CFI and RMSEA. For a small sample size (total *N* < 300), Chen (2007) recommended that a change of lower than 0.005 in CFI and a change of lower than 0.010 in RMSEA would indicate measurement invariance. Table 4 presents detailed statistical information of measurement invariance.

#### Between network validity

The between-network validity was examined by testing the association between the CAAS-SF dimensions and self-esteem using Pearson’s correlation analysis and a path analysis *via* structural equation modeling.

## Results

Table 1 presents descriptive statistics, reliabilities, and bivariate correlations of the four dimensions of career adaptability and their correlations with self-esteem as an external measure to assess the external validity of the CAAS-SF. Reliabilities of this scale and its subscales are acceptable for research purposes (ranging from 0.68 to 0.91 with an average Cronbach alpha value of 0.83). Reliability of the career confidence subscale in girls with SEN needs more attention and work. See the discussion part for our detailed discussion of this point.

**Table 1:** Descriptive statistics and bivariate correlations of career adaptability and self-esteem.

Variables	*M*	*SD*	α	1	2	3	4	5	6
**Total sample of SEN students**									
1. Concern	9.06	2.87	0.914	-	0.464**	0.658^**^	0.544^**^	0.814^**^	0.586^**^
2. Control	10.02	2.44	0.797		-	0.613^**^	0.635^**^	0.797^**^	0.534^**^
3. Curiosity	9.94	2.61	0.839			-	0.709^**^	0.891^**^	0.537^**^
4. Confidence	10.12	2.29	0.682				-	0.849^**^	0.474^**^
5. CAAS-SF-total	39.19	8.56	0.914					-	0.639^**^
6. Self-esteem	9.48	2.72	0.820					-	
**Male SEN students**									
1. Concern	8.97	2.94	0.935	-	0.511^**^	0.726^**^	0.561^**^	0.832^**^	0.564^**^
2. Control	10.11	2.53	0.809		-	0.681^**^	0.661^**^	0.823^**^	0.603^**^
3. Curiosity	9.95	2.69	0.844			-	0.745^**^	0.921^**^	0.532^**^
4. Confidence	10.25	2.36	0.722				-	0.854^**^	0.514^**^
5. CAAS-SF-total	39.32	9.03	0.926					-	0.645^**^
6. Self-esteem	9.54	2.79	0.846						-
**Female SEN students**									
1. Concern	9.20	2.81	0.873	-	0.376^**^	0.516^**^	0.525^**^	0.782^**^	0.645^**^
2. Control	9.89	2.32	0.771		-	0.464^**^	0.572^**^	0.739^**^	0.403^**^
3. Curiosity	9.94	2.50	0.831			-	0.632^**^	0.821^**^	0.565^**^
4. Confidence	9.90	2.19	0.594				-	0.841^**^	0.403^**^
5. CAAS-SF-total	39.00	7.82	0.883					-	0.644^**^
6. Self-esteem	9.29	2.63	0.776						-

The sub-scale of confidence in female students (*n* = 70) has α = 0.70 if confidence items 1 was deleted. See the discussion part for our elaboration.

** *p* ≤ 0.001.

Table 2 presents the model fit results, and Table 3 shows detailed factor loadings of the three models. The general factor model showed a poor fit with our data (CFI = 0.788, TIL = 0.741, RMSEA = 0.13, SRMR = 0.082). By contrast, both the four-correlated factor model and second-order model showed excellent fit (CFIs = 0.973, 966, TLIs = 0.963, 955, RMSEAs = 0.049, 0.054, and SRMRs = 0.043, 0.047), and the factor loadings in these two models ranging from 0.530 to 0.988. The four correlated factors model fits slightly better than the second-order model.

**Table 2:** Model fit indices of the three alternative/competing models in Figure 1.

Model	Model description	χ^*2*^	*df*	CFI	TLI	RMSEA [90% CI]	SRMR
1	General factor	239.769	54	0.788	0.741	0.130 [0.117, 0.144]	0.082
2	Four correlated factors	71.63	48	0.973	0.965	0.049 [0.028, 0.068]	0.043
3	Second order model	79.821	50	0.966	0.955	0.054 [0.035, 0.072]	0.047

Maximum likelihood with robust standard errors (MLR) was used for running these CFA models as a commonly used estimation method. The number of observations was 203, excluding 1 participant who did not respond to any CAAS-SF items.

**Table 3:** Standardized factors loadings of the three alternative models.

Item No.	Item labels	Item statement	CFA model 1 General factor	CFA model 2 Correlated factors	CFA model 3	
			Unidimensional	Multidimensional	Second order factor	
			CAAS	Correlated factors	First order factors	Second order factors
C1	Concern1	Thinking about what my future will be like.	0.757	0.864	0.865	
C2	Concern2	Preparing for the future.	0.820	0.964	0.962	
C3	Concern3	Becoming aware of the educational and vocational choices that I must make.	0.761	0.831	0.832	
C4	Control1	Making decisions by myself.	0.678	0.801	0.813	
C5	Control2	Taking responsibility for my actions.	0.529	0.687	0.679	
C6	Control3	Counting on myself.	0.560	0.775	0.768	
C7	Curiosity1	Looking for opportunities to grow as a person.	0.822	0.854	0.843	
C8	Curiosity2	Investigating options before making a choice.	0.740	0.788	0.789	
C9	Curiosity3	Observing different ways of doing things.	0.709	0.753	0.766	
C10	Confidence1	Taking care to do things well.	0.652	0.680	0.680	
C11	Confidence2	Learning new skills.	0.646	0.688	0.688	
C12	Confidence3	Working up to my ability.	0.478	0.530	0.531	
		Mean value of factor loadings	**0.679**	**0.768**	**0.768**	0.747 (for Concern)
						0.795 (for Control)
						0.968 (for Curiosity)
						0.988 (for Confidence)

**Table 4:** Fit statistics of measurement invariance tests across gender based on (1) the correlated factors model and (2) the second-order factor model.

CFA Model	Model fit indices								Chi-squared difference test				
	χ^*2*^	*df*	*p*	RMSEA [90% CI]	CFI	TLI	SRMR	AIC	$\Delta \chi^{2}$	Δdf	*p*	Δ CFI	Δ RMSEA
**Correlated factors**													
Male (*n* = 131)	64.964	48	0.052	0.052 [0.017, 0.052]	0.976	0.968	0.047	3487.59					
Female (*n* = 70)	86.281	48	0.001	0.107 [0.072, 0.140]	0.870	0.822	0.077	2046.95					
Configural	149.666	96	0.000	0.075 [0.053, 0.095]	0.948	0.934	0.058	5534.54					
Metric	156.494	104	0.001	0.071 [0.050, 0.091]	0.949	0.936	0.060	5524.05	5.489	8	0.704	0.001	0.002
Scalar	161.840	112	0.001	0.067 [0.045, 0.086]	0.952	0.943	0.061	5511.95	3.879	8	0.868	0.003	0.001
Residual	188.303	124	0.000	0.072 [0.053, 0.090]	0.938	0.934	0.072	5523.05	25.582	12	0.012	0.014	0.011
**Second order factor**													
Male (*n* = 131)	61.098	47	0.081	0.048 [0.000, 0.075]	0.980	0.973	0.044	3484.57					
Female (*n* = 70)	87.200	47	0.000	0.111 [0.076, 0.144]	0.864	0.809	0.077	2048.95					
Configural	146.193	94	0.000	0.075 [0.053, 0.095]	0.950	0.929	0.055	5533.52					
Metric	151.262	102	0.001	0.069 [0.047, 0.090]	0.952	0.944	0.058	5522.03	4.098	8	0.848	0.002	0.006
Scalar	156.537	110	0.002	0.065 [0.042, 0.085]	0.955	0.946	0.065	5509.83	3.793	8	0.875	0.003	0.004
Residual	163.166	122	0.008	0.058 [0.035, 0.090]	0.960	0.957	0.061	5497.57	8.085	12	0.779	0.005	0.007

### Measurement invariance across gender

Based on the results of three alternative CFA models (see Figure 1) to explore the factor structure (see Table 2), we tested measurement invariance based on the four correlated factors model (Model 2 in Figure 1). The configural model showed an excellent fit, indicating the factor structure was invariant across gender. There was no significant difference in model fit between the configural and the metric models (ΔCFI = 0.001, ΔRMSEA = 0.002), as well as between the metric and scalar models (ΔCFI = 0.003, ΔRMSEA = 0.001), supporting the presence of metric and scalar measurement invariance (i.e., factor loadings and item intercepts were invariant across gender). Compared to the scalar model, the residual model showed a significant decrease in CFI (ΔCFI = 0.014), not supporting residual invariance. The results were similar when conducting measurement invariance tests across gender with the second-order model used in Maggiori et al.’s (2017) study as the unconstrained baseline model. Given the current sub-sample sizes of boys and girls are not equally distributed, we suggest further research consider increasing the sample size of girls with SEN purposefully to replicate the present study to verify the results.

### Between network validity

The correlation between self-esteem and the overall career adaptability for the total sample, boys and girls with SENare *r* = 0.64, *r* = 0.65 and *r* = 0.64 (*p*s ≤ 0.001), demonstrating a positive association between self-esteem and career adaptability. Self-esteem positively correlated with the four dimensions of career adaptability, ranging from *rs* = 0.47/0.51/0.40 (*p*s ≤ 0.001) to *rs* = 0.64/0.60/0.65 (*p*s ≤ 0.001) for the total sample/boys/girls. For detailed correlation coefficients, see Table 1. No significant correlation was found between gender and CAAS-SF/self-esteem (with *p*-values ranging from 0.291 to 0.992).

**Figure 2:**
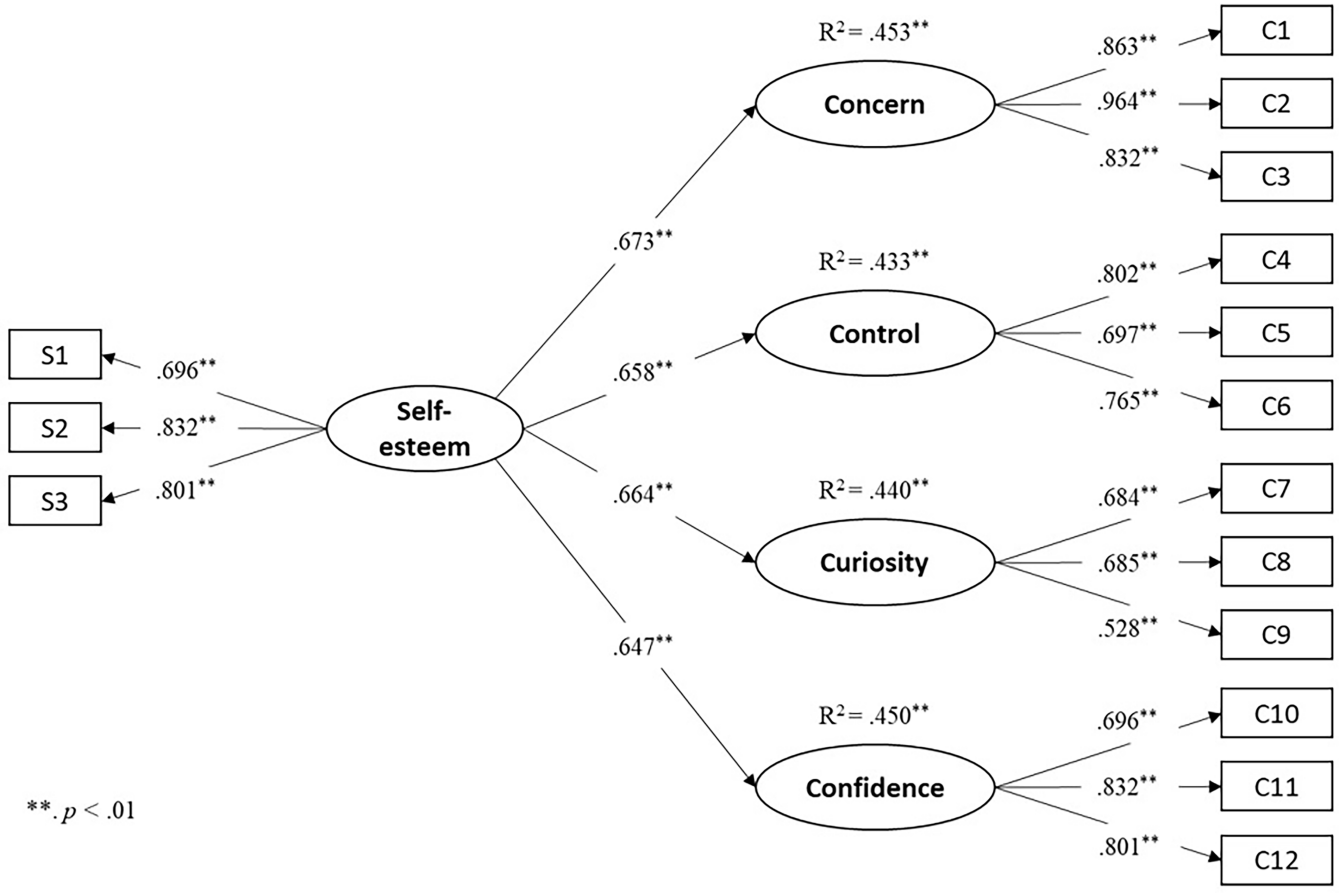
The SEM model with self-esteem as an independent variable and the four CASS dimensions as dependent variables (standardize coefficients).

Given the broader scope of self-esteem in assessing individuals’ perceptions of self-worth and the more specific features of the CAAS-SF in assessing individuals’ self-perceptions of career adaptability, we treated self-esteem as an independent variable and the four CAAS dimensions as the dependent variables. The SEM model showed good model fit indices (χ^2^ = 105.938, *df* = 80; *p* = 0.028; CFI = 0.978; TLI = 0.971; RMSEA [90% CI of RMSEA] = 0.040 [0.019, 0.059]; SRMR = 0.042). Self-esteem has a significant positive effect on each sub-dimension of career adaptability (see Figure 2). Future longitudinal studies would provide stronger empirical evidence shedding light on the reciprocal relationship between self-esteem and career adaptability.

## Discussion

This study took the initiative to validate the CAAS-SF with a sample of over 200 SEN students integrated into mainstream secondary schools in Hong Kong. The results showed that the 12-item CAAS has good psychometric properties in SEN students. Internal consistencies of the total scale and sub-scales are adequate for research purposes. The series of confirmatory factor analyses to test the three alternative models to explore the factor structure supported the multidimensionality of career adaptability for assessing career concern, career control, career curiosity, and career confidence. The one higher-order factor model was also consistent with previous research (Maggiori et al., 2017), suggesting that the four dimensions are nested in career adaptability. Measurement invariance tests supported the stability and invariance of this scale across boys and girls at the scalar level. Compared to Maggiori et al.’s (2017) study, we provided more model comparison results (e.g., one general factor, correlated factors, measurement invariance tests across both second-order and correlated factors models) for the audience to generate a better understanding of the factor structure with SEN students. Up to date, no such a single study has provided detailed results of the CAAS-SF psychometric properties with SEN students like the current one. Although a recent survey by Yuen and Chan (2022) included SEN students, their focus was on examining the relationship between a general factor of career adaptability with other variables (e.g., social connectedness and meaning in life).

Aside from providing empirical evidence to support the four-factor structure and reliabilities of sub-scales and the total CAAS-SF, one interesting finding in this study was about girls’ career confidence. By using three items to assess this dimension, we found there was a weak correlation (*r* = 0.128, *p* = 0.289) between confidence item 1 (*Taking care to do things well*) and item 3 (*Working up to my* ability). This correlation also influenced the scale reliability (α = 0.59, about 0.6). However, in social science, Churchill Jr (1979) suggested that a Cronbach’s alpha value of 0.6 is acceptable. By excluding confidence item 1, this scale with two items has better reliability (α = 0.70). Drost (2011, p. 114) commented: “a satisfactory level of reliability depends on how a measure is being used … reliabilities of 0.70 or higher will be sufficient.” Given that we aimed to compare the results by excluding no item from the original CAAS-SF with previous research (e.g., Maggiori et al., 2017), also given that “in social science, the acceptable α value is 0.60” suggested by Mohamad et al. (2015, p. 165), we kept all items in the sub-scale of confidence in our CFA, measurement invariance and path model analyses. Supplementary CFA in male and female students also found that the correlated factors model (Model 2 in Figure 1) has a much better fit to the data than the general factor model (Model 1 in Figure 1). Consistent with the total sample results, factor loadings are statistically significant and adequate for research purposes. The average scores of factor loadings found in boys and girls (both are larger than 0.70) are similar to that of the total sample. Considering these supplementary validity results, the Cronbach’s alpha value for the career confidence sub-scale in female students may need to be interpreted cautiously (without a superficial judgment). The model fit of boys appears better than that of girls. We should note that the model fit of girls seems barely acceptable at CFI = 0.87 (see Hayes, 1997; Ebesutani et al., 2011). We examined mean differences of the four dimensions of CAAS-SF and the total score of this scale between boys and girls and found no significant gender differences. Again, the smaller sample size of girls (*n* = 70) in this study, compared to that of boys (*n* = 131), need more attention and caution in interpreting the results. Future studies by purposefully increasing the sample size of female secondary students with special educational needs would help verify what we found in this study, especially the confidence sub-scale.

Using self-esteem as an external variable to examine the external validity of the CAAF-SF, we found self-esteem positively and significantly correlated with the four dimensions of career adaptability. This pattern appears consistent across the total sample, boys and girls (Mean correlation coefficients = 0.53, 0.55, 0.50, respectively). The results also align with previous research, although they used a typically developed sample of participants (Ismail et al., 2016). The additional path model (Figure 2) of using self-esteem (students’ general perception of self-worth) as a self-resource predictor of the four relatively specific dimensions of SEN students’ perceived career adaptability shows similar predictive effects of self-esteem on the four dimensions (with R square values ranging from 0.43 to 0.45).

### Theoretical and practical implications

Adaptability is a core/metacompetence proposed in career construction theory (Savickas, 2005, 2013). This study enriches career construction theory by adding valuable data from a sample of students with special educational needs across over 10 secondary schools in Hong Kong. This study validates the Chinese version of CAAS-SF among this vulnerable group of students.

The post-school transition period for SEN students has been found to much more challenges and difficulties (e.g., lower rates of completion of post-secondary education, lower participation in communities, unemployment) compared to their typically developing peers (Flexer, 2008; Sin and Yang, 2018; Lindsay et al., 2019). Identifying the population of SEN students’ features of career adaptability would be part of the prerequisites for developing effective/tailor-made career guidance and counseling to support their post-school outcomes and transition success (e.g., Test et al., 2009; Sprunger et al., 2018; Talapatra et al., 2019). Aside from supporting students’ regular learning activities, schools are committed to developing life-planning education, cultivating students’ career-planning and goal-setting abilities, and connecting their academic skills and interests to various career paths. Practically, the short form of CAAS is more feasible and manageable for school-based career counselors and life planning practitioners to assess students’ career adaptability resources for providing personalized and productive career guidance and life planning.

This study has implications for educators, school-based career guidance coordinators, and career practitioners. We found positive associations between the subdimensions of career adaptability and self-esteem. The results showed that self-esteem could explain 43 to 45% variance in career adaptability dimensions, suggesting self-esteem enhancement (e.g., Pope et al., 1988) would be one of the approaches contributing to SEN students’ career adaptability. Researchers also found that social support and social skills training effectively promote self-esteem (e.g., Chang et al., 2018; Seema and Kumar, 2018). A recent systematic review by Bang et al. (2022) argued that “adolescence is the period of self-concept formation; in particular, the quality of relationships is an important factor in forming self-concept” (p.36; see also Sebastian et al., 2008). School teachers can work closely with career guidance coordinators to design learning activities and career intervention programs tailor-made to cultivate the career adaptability of SEN students. Together with the CAAS-SF validated in this study and the positive association identified between career adaptability and self-esteem for developing productive school-based support for students’ post-school transition success, investigations of other variables, such as self-regulation, academic engagement, academic persistence, academic satisfaction, core self-evaluations, vocational identity would also be needed in helping students’ career construction (e.g., Merino-Tejedor et al., 2016; Wilkins-Yel et al., 2018; Kirchknopf, 2020; Sou et al., 2022).

### Limitations and future directions of research

Given the cross-sectional feature of this study, repeated measures of the CAAS-SF across multiple time points would provide valuable evidence of the stability of this instrument in assessing the career adaptability and personal resources of SEN students. Future studies can also improve the number of girls for more comparative purposes of the factor structure of the CAAS-SF. In this study, the sample size of girls with SEN was 70. Model fit indices among female students were not as good as for boys. This result may be influenced by the relatively small sample size of the female data. Further studies by increasing sample sizes or using equivalent sample sizes of male and female SEN students are anticipated to verify the results found in this study. However, we would also draw the readers’ attention to that the argument of the sample size of SEN students (compared to typically developing ones) may need to be considered given that there are often only several students with usually mild special educational needs integrated into regular/inclusive classrooms (Sin and Yang, 2018). Based on a commissioned report of a survey of 230 Hong Kong schools, most of the 192 participating schools (118 primary and 74 secondary) have less than 10% of SEN students (Sin et al., 2012).

Aside from exploring possibilities to enhance sample sizes, comparative studies between SEN and typically-developed students across cultures are anticipated to bring more insights into researchers’ and practitioners’ good understanding of the characteristics of career adaptability of SEN students. Doing so contributes to designing more tailor-made career counseling support to cultivate SEN students’ career adaptability and non-SEN counterparts. As a previous meta-analysis (Rudolph et al., 2017a, p. 17) showed, “career adaptability is significantly associated with measures of adaptivity, adapting responses, and various adaptation results (e.g., career/job/school satisfaction, affective organizational commitment, employability, promotability, life satisfaction).” The CAAS-SF validated in this study has important practical implications for future studies to evaluate school-based career guidance and life planning education for SEN and their typically-developing peers. These evaluation results would be valuable recourses for school stakeholders’ development of productive career guidance activities to support students with diverse needs in Hong Kong and other countries/regions. As Wong (2022) argued in a review of career adaptability, given that this contract was typically developed in Western cultures, cross-cultural empirical studies, as well as appropriate interpretations of career adaptability concerning local contexts of different research designs, need to be recognized and strengthened.

Given different types of special educational needs, future research by adopting a range of qualitative research approaches (e.g., individual-based interviews, focus group interviews, cross-sectional case studies, longitudinal case studies) would have their unique and salient roles in exploring the career adaptability of each type of SEN students in an in-depth way. The collective efforts of diverse research methods would lend informative recourses for personalized career guidance and life planning education. In Hong Kong, the number of SEN types is not equally distributed, as SpLD, ADHD, and ASD are three major types of SEN consistently identified in school settings. In comparison, the sizes of the other six types of SEN students appear to be considerably small (Legislative Council of Hong Kong, 2019). Future endeavors to recruit more SEN students in the six types are worthwhile to replicate the current study and examine the stability of the CAAS-SF across different types of SEN students. One positive alternative to recruiting similar sample sizes for comparison purposes would be to use innovative assessment tools developed recently in a post-school transition training programme to SEN school leavers based on the CAAS-SF (e.g., game-based assessment of CAAS, Sin et al., 2022) for expanding the scope of using a self-reported survey to assess career adaptability across different disabilities. Case study design (Flyvbjerg, 2011) also has great potential to explore and compare the career adaptability of different types of disabilities. Only recently could we find limited qualitative research on career adaptability among parents of children with autism spectrum disorder (Ozdemir and Koç, 2022), migrant workers (Ennerberg and Economou, 2022; Jones et al., 2022), newly graduated nurses (Zhang et al., 2022), and people with refugee backgrounds (Abkhezr et al., 2022). Qualitative research on the career adaptability of SEN students is also apparently lacking and calling for further explorations.

## Conclusion

Consistent with previous research examining the CAAS-SF in typically developed students and adults in the workplace, this study provides empirical evidence suggesting this instrument is also appropriate to assess the multidimensional career adaptability of students with special educational needs in Hong Kong. The internal structure result shows the four-factor solution (i.e., considering the separable features of career concern, control, curiosity, and confidence) suits the data best. With reliable CAAS-SF, early identification of SEN students’ career adaptability in schools would contribute to developing appropriate career guidance and life planning activities to assist in the post-school transition and career development of students with special education needs. Further mixed-research methods are anticipated to bring more insights into career assessment and support to career adaptability of the population of SEN students.

## Data availability statement

The raw data supporting the conclusions of this article will be made available by the authors, without undue reservation.

## Ethics statement

The studies involving human participants were reviewed and approved by Human Research Ethics Committees, The Education University of Hong Kong. Written informed consent to participate in this study was provided by the participants’ legal guardian/next of kin.

## Author contributions

LY proposed the study, conceptualized it, and designed the survey study with the consent and support of Prof. Mark L. Savickas (MS) to extend the CAAS-SF to SEN students. LY co-collected the data, wrote and finalized the complete draft. KS co-conceptualized this study, co-contributed to data collection and resources for the manuscript. MS co-conceptualized the design of this study in SEN students. LY and MS worked collectively to address all comments and finalize the manuscript. All authors contributed to the article and approved the submitted version.

## Funding

This project was supported by GRF (186105/15H) and funded by the research grant committee of Hong Kong to LY and KS.

## Conflict of interest

The authors declare that the research was conducted in the absence of any commercial or financial relationships that could be construed as a potential conflict of interest.

## Publisher’s note

All claims expressed in this article are solely those of the authors and do not necessarily represent those of their affiliated organizations, or those of the publisher, the editors and the reviewers. Any product that may be evaluated in this article, or claim that may be made by its manufacturer, is not guaranteed or endorsed by the publisher.

## Footnotes

^1^Intellectual Disability, Autism Spectrum Disorder, Attention Deficit/ Hyperactivity Disorder, Specific Learning Difficulties, Physical Disability, Visual Impairment, Hearing Impairment, Speech and Language Impairment, and Mental Illness. Source from https://sense.edb.gov.hk/en/types-of-special-educational-needs/

## Appendix

**Table A.1:** Standardized factors loadings of the general factor and correlated factors models (Models 1 and 2 in Figure 1) for male and female SEN students.

Item no.	Item labels	Item statement	Male SEN students		Female SEN students	
			CFA model 1	CFA model 2	CFA model 1	CFA model 2
			Unidimensional	Multidimensional	Unidimensional	Multidimensional
			CAAS	Correlated factors	CAAS	Correlated factors
C1	Concern1	Thinking about what my future will be like.	0.82	0.92	0.63	0.75
C2	Concern2	Preparing for the future.	0.85	0.98	0.77	0.94
C3	Concern3	Becoming aware of the educational and vocational choices that I must make.	0.81	0.83	0.67	0.82
C4	Control1	Making decisions by myself.	0.71	0.82	0.64	0.78
C5	Control2	Taking responsibility for my actions.	0.56	0.69	0.48	0.66
C6	Control3	Counting on myself.	0.61	0.79	0.45	0.75
C7	Curiosity1	Looking for opportunities to grow as a person.	0.84	0.86	0.80	0.81
C8	Curiosity2	Investigating options before making a choice.	0.77	0.80	0.66	0.78
C9	Curiosity3	Observing different ways of doing things.	0.73	0.76	0.66	0.78
C10	Confidence1	Taking care to do things well.	0.65	0.69	0.68	0.68
C11	Confidence2	Learning new skills.	0.67	0.73	0.58	0.56 (*p* = 0.001)
C12	Confidence3	Working up to my ability.	0.48	0.57	0.42 (*p* = 0.001)	0.39 (*p* = 0.018)
		Mean value of factor loadings	**0.71**	**0.79**	**0.62**	**0.72**

For factor loadings without adding specific *p* values to them, all of them are with *p* < 0.001. Fit indices of the correlated factors model for the two sub-groups of students have been listed in Table 4 in the main text, as such we did not repeat here.
